# One-Year Outcomes of CGuard Double Mesh Stent in Carotid Artery Disease: A Systematic Review and Meta-Analysis

**DOI:** 10.3390/medicina60020286

**Published:** 2024-02-08

**Authors:** Konstantinos Tigkiropoulos, Spyridon Nikas, Manolis Ampatzis-Papadopoulos, Katerina Sidiropoulou, Kyriakos Stavridis, Dimitrios Karamanos, Ioannis Lazaridis, Nikolaos Saratzis

**Affiliations:** 1 Division of Vascular Surgery, 1st Surgical Department, Faculty of Health Sciences, Papageorgiou General Hospital, Aristotle University, 56403 Thessaloniki, Greece; 2 Department of Radiology, Papageorgiou General Hospital, 56403 Thessaloniki, Greece

**Keywords:** CGuard, carotid artery stenting, carotid artery disease, dual layer mesh stents, systematic review and meta-analysis

## Abstract

*Background*: Prospective single and multicenter studies have shown improved outcomes of patients who underwent carotid artery stenting with the novel CGuard dual-layer mesh stent at 1 year. *Objectives*: The aim of this study is to conduct a systematic review and meta-analysis of all published studies to assess 1-year efficacy and outcomes of CGuard in patients with carotid stenting. Methods: A systematic search was performed. All studies enrolling at least 20 patients were included in our analysis. The primary endpoints were death (all-cause, cardiovascular and ipsilateral stroke-related death) and stroke rate at 1 year. The secondary endpoint was in-stent restenosis at 1 year. *Results*: The final analysis included 1709 patients. The one-year all-cause mortality rate was 2.97% (39/1699, 95% CI: 1.26–6.86%, I^2^ = 67%, t^2^ = 0.3442, *p* < 0.01), cardiovascular-related death was 0.92% (10/1616, 95% CI: 0.35–2.39%, I^2^ = 34%, t^2^ = 0.2302, *p* = 0.18), and ipsilateral stroke-related death was 0.3% (1/1649, 95% CI: 0.1–0.87%, I^2^ = 0%, t^2^ = 0, *p* = 0.69). The one-year ipsilateral stroke rate was 1.21% (16/1649, 95% CI: 0.58–2.5%, I^2^ = 28%, t^2^ = 0.1433, *p* = 0.23), transient ischemic attacks (TIAs) rate was 1.78% (19/1149, 95% CI: 1.11–2.84%, I^2^ = 0%, t^2^ = 0, *p* = 0.69), and total composite 1-year stroke/TIA rate was 2.97% (32/1149, 95% CI: 1.84–4.77%, I^2^ = 0%, t^2^ = 0, *p* = 0.41). The in-stent restenosis rate at 1 year was 1.06% (13/1653, 95% CI: 0.48–2.34%, I^2^ = 28%, t^2^ = 0.2308, *p* = 0.22). *Conclusions*: This meta-analysis shows that CAS with CGuard is safe with minimal neurological adverse events and in-stent restenosis rate at 1 year.

## 1. Introduction

Dual-layer mesh stents are considered a novel type of stent for the treatment of carotid artery stenosis (CAS). Their main advantage compared to first-generation stents (FGS) is the prevention of plaque prolapse and dislodgement of atheromatous debris after their deployment. Several studies have shown a higher rate of neurological events (30–60%) with FGS post-procedurally [1,2,3,4]. Dual-layer mesh stents combine the mechanical properties of open and closed cell stents with the presence of a mesh wrapping the nitinol skeleton. The CGuard (Inspire MD, Tel Aviv, Israel) microNET self-expanding stent with an embolic protection system (EPS) is a DLMS that was introduced in Europe in 2015, while it is still under investigation in the United States of America [5]. The aim of this systematic review and meta-analysis is to report the clinical efficacy of the CGuard carotid stent in terms of death (all cause, cardiovascular-related, ipsilateral stroke-related), ipsilateral stroke and in-stent restenosis rate at 1 year.

## 2. Methods

This study was conducted based on a protocol that is available at the PROSPERO site (registration number CRD42023484052). The latest PROSPERO search for ongoing systematic reviews on this subject did not reveal relevant ongoing protocols (November 2023). Case series, cohort studies, case-control studies, randomized controlled trials (RCTs) and non-randomized studies of interventions (NRSIs) were included as well as records from congresses and conferences regarding such types of studies with a minimum of 20 patients. Regarding studies with comparison arms, the arm with the CGuard stent was isolated. Single case reports, narrative reviews, systematic reviews and editorials were excluded. Only studies published in the English language were included for analysis. A reasonable publication date filter was used (after 2015), which corresponds to the first published studies with the CGuard stent.

### 2.1. Inclusion–Exclusion Criteria

The participants include symptomatic patients with >50% of carotid artery stenosis and asymptomatic patients with a diagnosis of carotid artery stenosis >60% with a life expectancy >5 years. Total occlusion and tandem lesions were excluded as well as other conditions affecting the carotid arteries, such as dissection, traumatic thrombosis, pseudoaneurysm or fibromuscular dysplasia (FMD). Rescue intervention cases for acute and evolving stroke were also excluded from the analysis.

### 2.2. Data Search

A full electronic database search was conducted. The databases searched were PubMed/MEDLINE, Scopus, ClinicalKey, Cochrane/CENTRAL and LILACS for published studies. The last search date was on 4 December 2023. A further search into registries of clinical trials on ClinicalTrials.gov (accessed on 4 December 2023), European Drug Regulating Authorities Clinical Trial Database and the WHO International Clinical Trials Registry Platform to identify ongoing and unpublished trials was conducted. The Chinese Clinical Trial Registry was not searched. The references of relevant studies were also searched (snowballing and citation searching) among those that were first screened against the eligibility criteria. From these, potentially relevant studies were also screened against the eligibility criteria. The archives of major conferences were also searched. Further searching via common search engines (i.e., Google) was conducted to identify relevant grey literature studies (i.e., via ResearchGate, Google Scholar, the InspireMD site).

A PubMed/MEDLINE search string was developed as follows: (“c-guard” OR cguard OR “c guard” OR “micro-net” OR micronet OR “mesh-covered” OR “mesh covered” OR “micromesh-covered” OR “micro mesh covered” OR “double mesh stent” OR “double-mesh stent” OR “dual-layered stent” OR DLS OR “dual layered stent” OR “dual layer stent” OR “dual-layer stent” OR “second generation carotid stent” OR “double layer stent” OR “double-layer stent” OR “double-layered stent” OR “double layered stent”) AND (CAS OR “carotid artery stenosis” OR “carotid artery stenting” OR “carotid artery disease” OR “carotid angioplasty” OR “carotid artery Revascularization” OR “carotid stent”). This search string was adequately adapted for the other databases.

### 2.3. Data Collection

A reference manager software was used (Mendeley). Two independent reviewers (KT and SN) ran the prespecified search algorithms. After deduplication, titles and abstracts were screened, and full-text articles were retrieved and screened against the eligibility criteria. Studies that were not available as full-text publications were excluded. A third adjudicator (M A-P) solved discrepancies between the two reviewers and concluded which final studies should be included. The study selection process was recorded and visually presented with a complete PRISMA (Preferred Reporting Items for Systematic Reviews and Meta-analyses) flow diagram.

The same independent reviewers (KT and SN) extracted the data from the selected studies using a prespecified form in Microsoft Excel that was developed by the lead author and pilot-tested using a random sample. Disagreement between the two reviewers was solved by the aforementioned adjudicator (M A-P). In the case of absent key information from the full text, the original authors were contacted via e-mail.

### 2.4. Critical Appraisal

This systematic review includes single-arm studies like case series (proportional meta-analysis). Risk-of-bias assessment tools are not widely used for these types of studies. We used the Joanna Briggs Institute (JBI) critical appraisal tool for case series instead, which employs a qualitative approach for study inclusion [6].

### 2.5. Statistical Analysis

The total number of included patients was calculated as well as the number of patients with every demographic or intervention characteristic. Forest plots (cumulative 12-month results) were generated to calculate the mean value for each separate studied outcome, and the respective percentage/incidence was extracted so that it could be compared to the findings of various studies in the literature. The 95% confidence intervals (95% CI) and heterogeneity of studies (I^2^ as well as between-studies variance [t^2^]) for each separate outcome were calculated, and the statistical significance of heterogeneity was studied and reported as important when *p* < 0.05. We performed a proportional meta-analysis. Because the CI limits in such analyses would probably fall outside the [0,1] interval, which would be illogical for proportions, we employed the logit transformation of the data and then a back-transformation before running the meta-analysis with the inverse variance method. The software used for this statistical analysis was the R programming language for statistical analysis (Posit, Boston, MA, USA, v. 4.2.1) and its development environment RStudio (v. 2022.07.1) using the package “meta” to calculate effect estimates and to produce forest plots. Both the fixed- and the random-effects models were utilized and presented, and the prediction interval was provided. No subgroup or sensitivity analyses were planned for this meta-analysis. An assessment of publication bias with classic methods (funnel plots, Egger’s test, Begg’s) is not advisable with proportional meta-analyses. As a consequence, we conducted a qualitative respective assessment.

### 2.6. Endpoints–Definitions

The primary endpoints were death (all-cause, cardiovascular and ipsilateral stroke-related death) and stroke rate at 1 year. The secondary endpoint was in-stent restenosis at 1 year. Cardiovascular death was defined as death due to myocardial infarction and cardiac failure during follow-up. Stroke-related death was defined as death due to stroke during follow-up. In-stent restenosis was defined as the presence of stenosis >70% or occlusion on ultrasonographic examination after carotid stenting beyond the 30th postoperative day (POD) until 1 year.

## 3. Results

**Figure 1:**
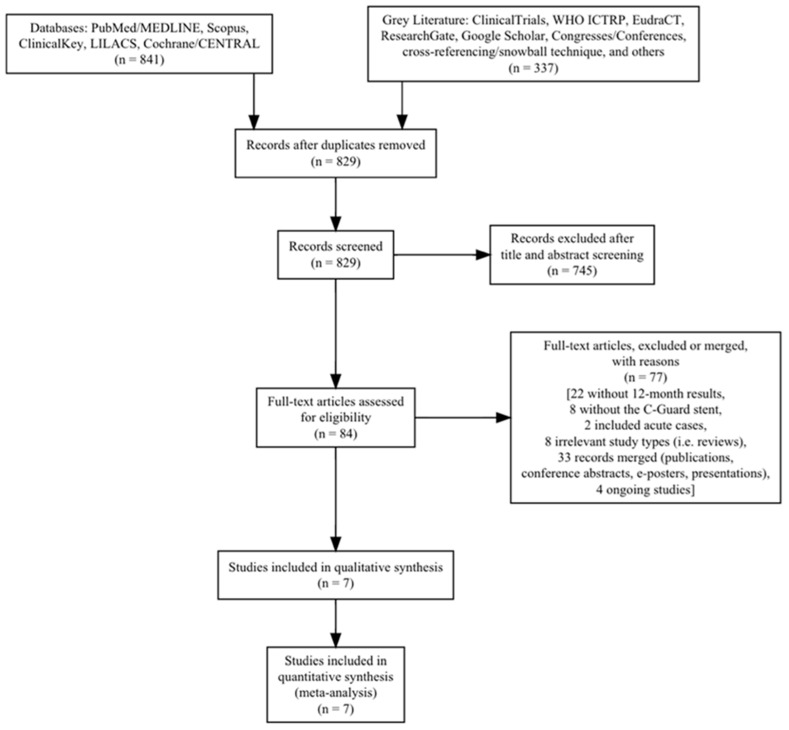
Preferred Reporting Items for Systematic Reviews and Meta-Analyses (PRISMA) flow chart.

A total of 1178 records were found, and after deduplication of 349 records, a total of 829 records were screened via their title and abstract. A total of 745 records were excluded as irrelevant, leaving 84 articles to be assessed as full-text publications and screened against the eligibility criteria. Seventy-seven records were further excluded (thirty-three potential same-study reports were merged together, and four ongoing studies were identified [5,7,8,9] (Table 1)), leaving seven studies for inclusion (Table 2) [7,10,11,12,13,14,15]. The search result is presented in a Preferred Reporting Items for Systematic Reviews and Meta-Analyses (PRISMA) flow chart (Figure 1).

### Characteristics of Included Studies

The detailed characteristics of the included studies, regarding patient demographics and intervention characteristics, are presented in Table 3. All included studies had similar indications for intervention, and most were undertaken in academic hospitals from experienced interventionists. Preoperative and postoperative use of antiplatelet therapy was also similar between studies. The total number of participants was 1709 (1742 arteries, mean age 71.63 years, 71.1% men). The pooled analysis resulted in an overall 99.4% rate of 1-year follow-up. The demographic characteristics and comorbidity distribution were comparable between the studies with some exceptions: Symptomatic patients varied from less than 10% to almost 60% of the cohort. Especially notable is the discrepancy of the use of embolic protection devices (EPDs). Most studies used, almost universally, EPDs mostly of the distal type except Tigkiropoulos et al. [14] who reported the use of an EPD in 5.3% of the patients. Nevertheless, the studies had comparable results, both mid-term and long-term.

All included studies reported 1-year overall mortality and in-stent restenosis rates, others as a dichotomous and others as a survival variable. Earlier records with their 30-day results were also retrieved, and the in-between time-period (30 days to 12 months) results were calculated when needed. The assessment of methodologic quality of studies included in our meta-analysis is presented in Table 4.

## 4. Findings

**Figure 2:**
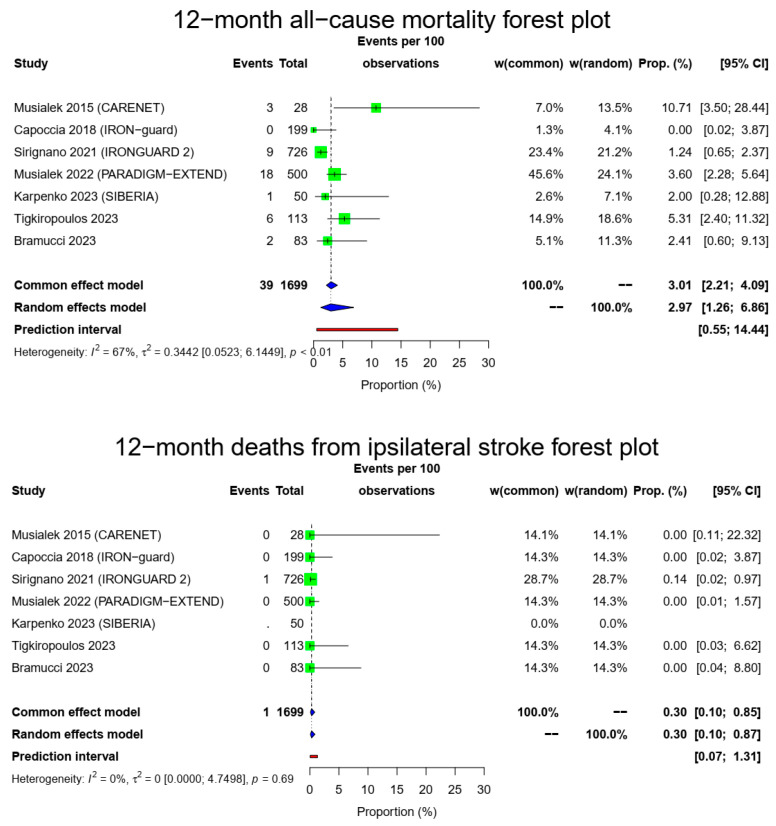

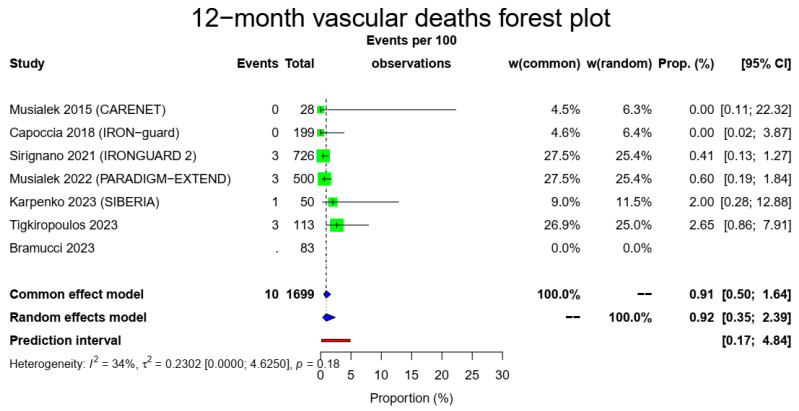
All-cause mortality, vascular deaths and deaths from ipsilateral stroke forest plots (cumulative 12-month results). Both common- and random-effect models are provided together with the prediction interval for each forest plot [7,10,11,12,13,14,15].

The pooled 1-year all-cause mortality rate was 2.97% (39/1699, 95% CI: 1.26–6.86%, random-effects model). This result had a relatively high heterogeneity (I^2^ = 67%, t^2^ = 0.3442, *p* < 0.01), most likely due to the higher death rate of the CARENET study^10^. There was only one death due to ipsilateral stroke in the whole cohort (1/1649, 0.3%, 95% CI: 0.1–0.87%, random-effects model, I^2^ = 0%, t^2^ = 0, *p* = 0.69), which occurred during the first 30 days, while the cardiovascular-related death rate was also low (10/1616, 0.92%, 95% CI: 0.35–2.39%, random-effects model, I^2^ = 34%, t^2^ = 0.2302, *p* = 0.18). The Figure 2 forest plot demonstrates 1-year all-cause, cardiovascular and ipsilateral stroke death.

**Figure 3:**
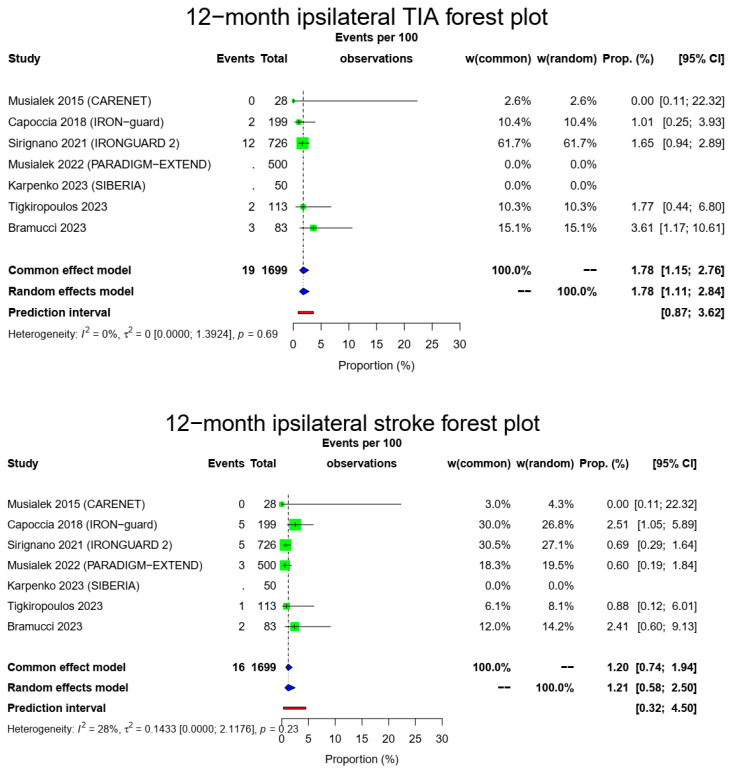

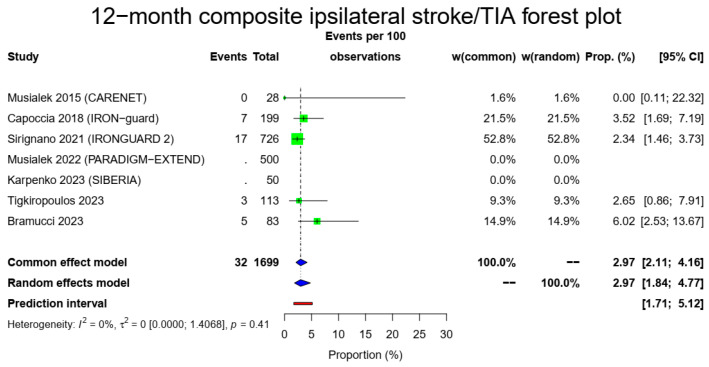
Ipsilateral stroke, TIA and composite ipsilateral stroke/TIA forest plots (cumulative 12-month results). Both common- and random-effect models are provided together with the prediction interval for each forest plot [7,10,11,12,13,14,15].

The pooled 1-year ipsilateral stroke rate was 1.21% (16/1649, 95% CI: 0.58–2.5%, random-effects model) with a low heterogeneity (I^2^ = 28%, t^2^ = 0.1433, *p* = 0.23). Of those, only three occurred after the 30-day time point, meaning that most ipsilateral strokes were during the early postoperative period. There were 19/1149 12-month ipsilateral TIAs (1.78%, 95% CI: 1.11–2.84%, random-effects model, I^2^ = 0%, t^2^ = 0, *p* = 0.69) with most of those occurring during the first month, and a total composite 1-year stroke/TIA rate of 2.97% (32/1149, 95% CI: 1.84–4.77%, random-effects model, I^2^ = 0%, t^2^ = 0, *p* = 0.41). The Figure 3 forest plot demonstrates 1 year of ipsilateral stroke, TIAs and combined stroke/TIAs.

**Figure 4:**
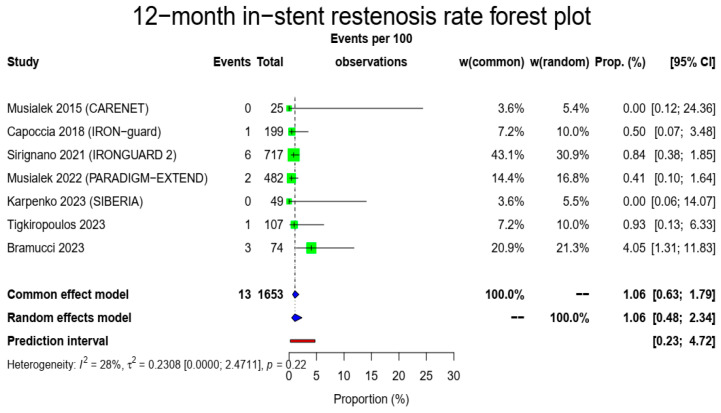
In-stent restenosis rate forest plot. Both common- and random-effect models are provided together with the prediction interval [7,10,11,12,13,14,15].

There were 1653 patients alive and available for imaging follow-up to evaluate 1-year in-stent restenosis. The pooled in-stent restenosis rate was 1.06% (13/1653, 95% CI: 0.48–2.34%, random-effects model) with a low heterogeneity (I^2^ = 28%, t^2^ = 0.2308, *p* = 0.22). The Figure 4 forest plot demonstrates the in-stent restenosis at 1 year.

## 5. Discussion

The main findings from this systematic review and meta-analysis evaluating the efficacy and outcomes of patients with carotid artery stenosis treated with the CGuard DLMS at 1 year are the following: (1) ipsilateral stroke-related death at 1 year was <1%; (2) pooled 1-year ipsilateral stroke/TIAs/combined stroke–TIAs rates were 1%, 1.7% and 2.8%, respectively; and (3) in-stent restenosis rate at 1 year was 0.8%.

Carotid artery stenting has emerged as an alternative to carotid endarterectomy for atherosclerotic carotid lesions, especially in high-risk patients with concomitant comorbidities. In a meta-analysis by Muller et al. [16], CAS periprocedural adverse events (major stroke, death) were statistically non-significantly increased compared to carotid endarterectomy (OR 1.36, 95% CI 0.97 to 1.91; *p* = 0.08, I^2^ = 0%; seven trials, 4983 participants; high-certainty evidence). Additionally, the risks for MI, access site hematoma and cranial nerve injury were lower compared to carotid endarterectomy. However, in symptomatic patients, CAS was associated with a significantly higher risk of periprocedural stroke and death [16].

First-generation carotid stents were made of stainless steel, cobalt alloy and nitinol [17]. Stainless steel is considered a strong material with a high radial force that prevents stent closure. However, it was associated with higher vessel wall injury, non-optimal wall apposition compared to nitinol stents and higher inflammatory reaction, promoting restenosis due to metal ion release [18,19]. Cobalt alloy stents provided good vessel wall apposition and had thinner struts, theoretically reducing restenosis [20]. Vajda et al., however, demonstrated a high in-stent restenosis rate in their study with the take-home message being close follow-up of the patients with this type of stent [21]. Nitinol is the most commonly used material for carotid stents nowadays. It is characterized by two unique properties: superelasticity that provides better apposition of the stent against the carotid wall and shape-memory properties [22].

Dual-layered mesh stents have been developed to minimize the risk of plaque protrusion and subsequent periprocedural stroke events. There are two types of DLMS available in the market: Roadsaver (Terumo Corp, Tokyo, Japan) and CGuard (Inspire MD, Tel Aviv, Israel). The efficacy of Roadsaver at 1 year has been evaluated in two studies [23,24]. Nerla et al. demonstrated no cerebrovascular events, and death was not related to the CAS procedure [23]. The CLEAR-ROAD study showed a 4.2% ipsilateral stroke rate and 7.5% restenosis rate at 1 year [24]. The ROADSAVER is a prospective, multi-center observational study that enrolled approximately 2000 patients across Europe, which evaluated the safety and efficacy of the Roadsaver stent at 30 days and 1 year. The study completion was July 2022, and we await the results for better analysis and evaluation of the Roadsaver carotid stent [25].

In our meta-analysis, CGuard has demonstrated a low rate of neurologic events at 1 year. The ipsilateral stroke rate is 1%, which is comparable to a previous patient-based meta-analysis showing a stroke rate of 1.6% and 0.26% in the Roadsaver group [26,27]. The results are considered acceptable compared to previous randomized trials where the ipsilateral stroke rate was 6% at 1 year [1]. However, no RCT has shown that stent type affects neurologic outcome [1,13]. In-stent restenosis is considered “The Achilles heel” regarding the efficacy of a carotid stent. CGuard has shown a low rate of ISR (0.8%) at 1 year. Its superiority against Roadsaver has been verified in a previous meta-analysis [26,27]. Mazurek et al. [27] showed that the ISR rate of the CGuard group was 0.34% compared to the Roadsaver group’s ISR rate of 7.16% (reduction of 6.82%, *p* < 0.001), whereas an in-patient-based meta-analysis by Stabile et al. [26] reported that the ISR rate of the CGuard group was 0.65% compared to 4% for the Roadsaver group (*p* = 0.007). A possible explanation could be the smaller size of the micromesh pores (CGuard 150–180 μm vs. Roadsaver 375–700 μm), reducing plaque rupture and prolapse through struts and their structural characteristics [14].

The midterm data regarding the mortality of patients who underwent CAS with DLMS are still limited due to the presence of a small number of observational studies. All-cause mortality is 2.3%, stroke ipsilateral mortality is <0.1%, and cardiovascular-related mortality is 0.8% at 1 year in this meta-analysis. All-cause mortality is similar compared to previous RCTs [1,28,29]. Nerla et al. [23] reported three deaths at the 12-month follow-up with the Roadsaver DLMS. Arterial disease in multiple vascular beds (heart, lower extremities) is frequent in patients with carotid artery stenosis, increasing their mortality after revascularization interventions [30,31,32].

## 6. Limitations

This meta-analysis is limited to the efficacy and outcomes of the CGuard DLMS at 1 year. Most of the studies are observational, prospective, single-center or multicenter, single-arm without a comparative study group and with differences in the volume of patients. Although observational studies have a lower level of evidence compared to randomized ones, the methodologic quality of the included studies in this meta-analysis was assessed, and they were considered as eligible. The meta-analysis results are based on seven published studies, a fact that limits the external validity and generalization of our results in all patients. Only a few appraisal tools are available to assess the methodologic quality of case series studies, and the Briggs Institute critical appraisal tool is the most known and used. The tool has not been designed based on the domain approach, critical appraisal questions are not “equal”, cut-off thresholds are not advised and should be decided by the reviewers themselves and the presentation of the results should be through a table. We presented the respective table, but we also set a cut-off threshold that was decided by two reviewers who assessed the methodologic quality of each included study independently. A sensitivity analysis and generation of diagrams, such as a funnel plot diagram, were not applicable due to the lack of a control group during the extraction of our meta-analysis results. Despite the fact that case studies that do not include a control group with negative results are expected to be concealed and not published, the known tests for assessing selective reporting bias were developed for comparative studies. Selective reporting bias was assessed qualitatively in this meta-analysis. The number of symptomatic patients included in the eligible studies ranged from less than 10% to almost 60%, which cannot exclude potential bias. However, the low heterogeneity regarding ipsilateral stroke and ISR rate make the meta-analysis results acceptable.

## 7. Conclusions

The present systematic review and meta-analysis suggests that CAS with the CGuard DLMS is safe with minimal neurological adverse events and in-stent restenosis rate at 1 year. However, larger RCTs and observational studies with a longer follow-up are necessary to establish its efficacy and superiority against the other types of carotid stents.

## Figures and Tables

**Table 1:** Ongoing studies: Information from merged records (journal publications, presentations, conference abstracts). * Results from those 500 patients are included in our meta-analysis [5,8,9].

Study	Country	Site(s)	Setting	Trial Identifier	Patients (Estimated)	Enrollment Phase	Already Published
**PARADIGM-EXTEND** [7]	Poland	Multi-center	Academic	NCT04271033	550	Recruiting	12-month results of up to 500 patients presented *
**C-GUARDIANS** [5]	Multiple countries	Multi-center	Company-sponsored	NCT04900844	316	Finished	30-day results, awaiting 12-month results
**OPTIMA** [8]	Multiple countries	Multi-center	Academic	NCT04234854	339	Finished	30-day results, awaiting 12-month results
**POLGUARD** [9]	Poland	Single-center	Academic	-	203	Finished	30-day results, awaiting 12-month results

**Table 2:** Information regarding included studies [7,10,11,12,13,14,15].

Study (First Author, Year, Acronym)	Country	Centers	Timing and Type	Source(s) of Information	All Patients	All Arteries	Patients Available for Follow-Up Studies
**Musialek 2015 (CARENET)** [10]	Germany, Poland	Multi-center	Prospective (cohort)	Publications, presentations	30	30	28
**Capoccia 2018** **(IRON-guard)** [11]	Italy	Multi-center	Prospective (cohort)	Publications, presentations, conference abstracts	200	200	199
**Sirignano 2021 (IRONGUARD 2)** [12]	Italy	Multi-center	Prospective (cohort)	Publications, presentations, conference abstracts	733	733	726
**Musialek 2022 (PARADIGM-EXTEND)** [7]	Poland	Multi-center	Prospective (cohort)	Publications, ClinicalTrials protocol, presentations, conference abstracts	500	533	500
**Karpenko 2023 (SIBERIA)** [13]	Russia	Single-center	Prospective (RCT)	Publications, ClinicalTrials protocol, presentations, conference abstracts	50	50	50
**Tigkiropoulos 2023** [14]	Greece	Single-center	Prospective (cohort)	Publications, individual patient data	113	113	113
**Bramucci 2023** [15]	Italy	Single-center	Retrospective (cohort)	Publications, individual patient data	83	83	83

**Table 3:** Patient and intervention characteristics of the included studies [7,10,11,12,13,14,15].

Study	All Patients	Patients with Follow-Up Available *	Males (%)	Arteries	Symptoms (%)	Age (m *± SD)*	HTN (%)	CAD (%)	DM (%)	PAD (%)	Smoking History (%) **	AF (%)	DLP (%)		EPD (%)	
														All EPD (%)	Proximal EPD (%)	Distal EPD (%)
**Musialek 2015 (CARENET)** [10]	30	28 (93.3%)	19 (63.3%)	30	10 (33.3%)	71.6 ± *7.6*	25 (83.3%)	8 (26.7%)	7 (23.3%)	NR	4 (13.3%)	NR	27 (90%)	30 (100%)	1 (3.3%)	29 (96.7%)
**Capoccia 2018 (IRON-guard)** [11]	200	199 (0.5%)	132 (66%)	200	17 (8.5%)	72.6 ± *7.09*	174 (87%)	68 (34%)	56 (28%)	NR	124 (62%)	NR	148 (74%)	200 (100%)	18 (9%)	182 (91%)
**Sirignano 2021 (IRONGUARD 2)** [12]	733	726 (99%)	516 (70.4%)	733	131 (17.9%)	73.03 ± *7.84*	622 (84.9%)	278 (37.9%)	264 (36%)	NR	429 (58.5%)	NR	552 (75.3%)	731 (99.7%)	138 (18.9%)	593 (81.1%)
**Musialek 2022 (PARADIGM-EXTEND)** [7] ***	500	500 (100%)	363 (72.6%)	533	299 (59.8%)	69.96 ± *8.14*	NR	152 (30.4%)	NR	NR	NR	68 (13.6%)	NR	533 (100%)	259 (48.6%)	274 (51.4%)
**Karpenko 2023 (SIBERIA)** [13]	50	50 (100%)	38 (76%)	50	16 (32%)	65 ± *7.5*	48 (96%)	39 (78%)	10 (20%)	15 (30%)	17 (34%)	NR	NR	50 (100%)	0 (0%)	50 (100%)
**Tigkiropoulos 2023** [14] ****	113	113 (100%)	82 (72.6%)	113	54 (47.8%)	70.14 ± *8.63*	91 (80.5%)	44 (38.9%)	42 (37.2%)	42 (37.2%)	66 (58.4%)	12 (10.6%)	89 (78.8%)	6 (5.3%)	0 (0%)	6 (100%)
**Bramucci 2023** [15] ****	83	83 (100%)	65 (78.3%)	83	30 (36.1%)	73 ± *13*	77 (92.8%)	32 (38.6%)	41 (49.4%)	NR	64 (77.1%)	14 (16.9%)	79 (95.2%)	81 (97.6%)	59 (72.8%)	22 (27.2%)
**Total (%)**	**1709**	**1699/1709 (99.4%)**	**1215/1709 (71.1%)**	**1742**	**557/1709 (32.6%)**	**71.63 *± 8.39***	**1037/1209 (85.8%)**	**621/1709 (36.3%)**	**420/1209 (34.7%)**	**57/163 (35%)**	**704/1209 (58.2%)**	**94/696 (13.5%)**	**895/1159 (77.2%)**	**1631/1742 (93.6%)**	**475/1631 (29.1%)**	**1156/1631 (70.9%)**

HTN: hypertension, CAD: coronary artery disease, DM: diabetes mellitus, PAD: peripheral arterial disease, smoking history: either current or former smokers, AF: atrial fibrillation, DLP: dyslipidemia, EPD: embolic protection device. * Patients who declined further follow-up or were lost to follow-up were excluded regarding the primary endpoints (per-protocol analysis). ** Some studies reported only current smokers, while others reported a smoking history in general. This could explain the discrepancy between studies. *** Data from the PARADIGM-EXTEND study were extracted from the latest record available. **** Based on individual patient data provided by the authors.

**Table 4:** Assessment of the methodological quality of the included studies using the Joanna Briggs Institute (JBI) critical appraisal tool for case series [7,10,11,12,13,14,15].

Study	Clear Inclusion Criteria	Standard, Reliable Measurement	Valid Identification Method	Consecutive Inclusion of Patients	Complete Inclusion of Patients	Clear Report of Participant Demographics	Clear Report of Patient Clinical Information	Clear Report of Outcomes/Follow Up	Clear Report of Presenting Site(s)’/Clinic(s)’ Demographic Information *	Appropriate Statistical Analysis **	Overall Appraisal
**Musialek 2015 (CARENET)** [10]	Yes	Yes	Yes	Yes	Unsure	Yes	Yes	Yes	Unsure	Not applicable	Include
**Capoccia 2018 (IRON-guard)** [11]	Yes	Yes	Yes	Yes	Yes	Yes	Yes	Yes	Unsure	Yes	Include
**Sirignano 2021 (IRONGUARD 2)** [12]	Yes	Yes	Yes	Yes	Yes	Yes	Yes	Yes	Unsure	Yes	Include
**Musialek 2022 (PARADIGM-EXTEND)** [7]	Yes	Yes	Yes	Yes	Yes	Yes	Yes	Yes	Unsure	Yes	Include
**Karpenko 2023 (SIBERIA)** [13]	Yes	Yes	Yes	Yes	Yes	Yes	Yes	Yes	Unsure	Yes	Include
**Tigkiropoulos** [14] **2023**	Yes	Yes	Yes	Yes	Unsure	Yes	Yes	Yes	Unsure	Not applicable	Include
**Bramucci 2023** [15]	Yes	Yes	Yes	No	Unsure	Yes	Yes	Yes	Unsure	Not applicable	Include

* A clear report of the presenting site(s)’ or clinic(s)’ demographic information (i.e., socioeconomic status of patients, access to healthcare, etc.) was not mentioned in any study; however, from the information about the country of origin and the setting and when regarding the nature of the disease, we assume that these results are comparable. ** Some case series did not perform any kind of statistical analysis since the absence of a comparison arm does not necessitate such analyses; therefore, an assessment is not applicable.

## Data Availability

Electronic databases were used for the extraction of data.

## References

[1] Hill M., Brooks W.H., Mackey A., Clark M.W., Meschia F.J., Morrish F.W., Mohr J.P., Rhodes J.D., Popma J.J., Lal K.B. (2012). Stroke after carotid stenting and endarterectomy in the Carotid Revascularization Endarterectomy versus Stenting Trial (CREST). Circulation.

[2] Bonati L.H., Jongen L.M., Haller S., Flach H.Z., Dobson J., Nederkoorn P.J., Macdonald S., Gaines P.A., Waaijer A., Stierli P. (2010). New ischemic brain lesions on MRI after stenting or endarterectomy for symptomatic carotid stenosis: A sub study of the International Carotid Stenting Study (ICSS). Lancet Neurol..

[3] Fairman R., Gray W.A., Scicli A.P., Wilburn O., Verta P., Atkinson R., Yadav J.S., Wholey M., Hopkins L.N., Raabe R. (2007). The CAPTURE registry-Analysis of strokes resulting from carotid artery stenting in the post approval setting: Timing, location, severity, and type. Ann. Surg..

[4] Bosiers M., de Donato G., Deloose K., Verbist J., Peeters P., Castriota F., Cremonesi A., Setacci C. (2007). Does free cell area influence the outcome in carotid artery stenting?. Eur. J. Vasc. Endovasc. Surg..

[5] Christopher M.D. 30-Day Results from the C-Guardians Pivotal Trial of the CGuard™ Carotid Stent System. Proceedings of the VIVA 2023.

[6] Munn Z., Barker T.H., Moola S., Tufanaru C., Stern C., McArthur A., Stephenson M., Aromataris E. (2020). Methodological quality of case series studies: An introduction to the JBI critical appraisal tool. JBI Evid. Synth..

[7] Musialek P. The 3 Micromesh Stents and Their Value in CAS: Do the Design Differences Matter? Update on Carotid and Other Uses of the MicroNet-Covered Stent (C-Guard). Proceedings of the Veith Symposium.

[8] Musialek P. OPTIMA Endovascular Exclusion of Consecutive Patient High-Risk Carotid Plaque Using the MicroNet Covered Stent (OPTIMA). ClinicalTrialsGov.ID. http://www.clinicaltrials.gov/study/NCT04234854.

[9] Szkolka L., Lyko-Morawska D., Balocco S., Bedkowski L., Buczek M., Medon E., Wolkowski M., Dryjski M., Kuczmik W. (2023). Vascular surgery study of the CGuard MicroNet-covered stent in patients with indication to carotid revascularization: POLGUARD. J. Cardiovasc. Surg..

[10] Schofer J., Musiałek P., Bijuklic K., Kolvenbach R., Trystula M., Siudak Z., Sievert H. (2015). Prospective, Multicenter Study of a Novel Mesh-Covered Carotid Stent: The CGuard CARENET Trial (Carotid Embolic Protection Using MicroNet). JACC Cardiovasc. Interv..

[11] Capoccia L., Sirignano P., Mansour W., Sbarigia E., Speziale F. (2018). Twelve-month results of the Italian registry on protected CAS with the mesh-covered CGuard stent: The IRON-Guard study. EuroIntervention.

[12] Sirignano P., Stabile E., Mansour W., Capoccia L., Faccenna F., Intrieri F., Ferri M., Saccà S., Sponza M., Mortola P. (2021). 1-Year Results From a Prospective Experience on CAS Using the CGuard Stent System: The IRONGUARD 2 Study. JACC Cardiovasc. Interv..

[13] Karpenko A., Bugurov S., Ignatenko P., Starodubtsev V., Popova I., Malinowski K., Musialek P. (2021). Randomized Controlled Trial of Conventional Versus MicroNet-Covered Stent in Carotid Artery Revascularization. JACC Cardiovasc. Interv..

[14] Tigkiropoulos K., Sidiropoulou K., Abatzis-Papadopoulos M., Lazaridis I., Saratzis N. (2023). 12-Month Outcomes of Carotid Artery Stenting with CGuard MicroNET-Covered Stent: A Single-Center Study in 113 Patients. Angiology.

[15] Bramucci A., Fontana A., Massoni C.B., Vecchiati E., Freyrie A., Tusini N. (2023). Dual-vs. single-layer stents for endovascular treatment of symptomatic and asymptomatic internal carotid artery stenosis. Cardiovasc. Revasc. Med..

[16] Müller M.D., Lyrer P., Brown M.M., Bonati L.H. (2020). Carotid artery stenting versus endarterectomy for treatment of carotid artery stenosis. Cochrane Database Syst. Rev..

[17] He D., Liu W., Zhang T. (2015). The Development of Carotid Stent Material. Interv. Neurol..

[18] Sheth S., Litvack F., Dev V., Fishbein M.C., Forrester J.S., Eigler N. (1996). Subacute thrombosis and vascular injury resulting from slotted-tube nitinol and stainless steel stents in a rabbit carotid artery model. Circulation.

[19] Li L., Pan S., Zhou X., Meng X., Han X., Ren Y., Yang K., Guan Y. (2013). Reduction of in-stent restenosis risk on nickel-free stainless steel by regulating cell apoptosis and cell cycle. PLoS ONE.

[20] Tanaka N., Martin J.B., Tokunaga K., Abe T., Uchiyama Y., Hayabuchi N., Berkefeld J., Rüfenacht D.A. (2004). Conformity of carotid stents with vascular anatomy: Evaluation in carotid models. AJNR Am. J. Neuroradiol..

[21] Vajda Z., Miloslavski E., Guthe T., Schmid E., Schul C., Albes G., Henkes H. (2010). Treatment of intracranial atherosclerotic arterial stenoses with a balloon-expandable cobalt chromium stent (Coroflex Blue): Procedural safety, efficacy, and midterm patency. Neuroradiology.

[22] Stoeckel D., Pelton A., Duerig T. (2004). Self-expanding nitinol stents: Material and design considerations. Eur. Radiol..

[23] Nerla R., Micari A., Castriota F., Miccichè E., Ruffino M.A., de Donato G., Setacci C., Cremonesi A. (2018). Carotid artery stenting with a new-generation double-mesh stent in three high-volume Italian centres: 12-month follow-up results. EuroIntervention.

[24] Bosiers M., Deloose K., Torsello G., Scheinert D., Maene L., Peeters P., Müller-Hülsbeck S., Sievert H., Langhoff R., Callaert J. (2018). Evaluation of a new dual-layer micromesh stent system for the carotid artery: 12-month results from the CLEAR-ROAD study. EuroIntervention.

[25] Kedev S., Müller-Hülsbeck S., Langhoff R. (2022). Real-World Study of a Dual-Layer Micromesh Stent in Elective Treatment of Symptomatic and Asymptomatic Carotid Artery Stenosis (ROADSAVER). Cardiovasc. Interv. Radiol..

[26] Stabile E., de Donato G., Musialek P., Deloose K., Nerla R., Sirignano P., Mazurek A., Mansour W., Fioretti V., Esposito F. (2020). Use of Dual-Layered Stents for Carotid Artery Angioplasty: 1-Year Results of a Patient-Based Meta-Analysis. JACC Cardiovasc. Interv..

[27] Mazurek A., Malinowski K., Rosenfield K., Capoccia L., Speziale F., de Donato G., Setacci C., Wissgott C., Sirignano P., Tekieli L. (2022). Clinical Outcomes of Second- versus First-Generation Carotid Stents: A Systematic Review and Meta-Analysis. J. Clin. Med..

[28] Reiff T., Eckstein H.H., Mansmann U., Jansen O., Fraedrich G., Mudra H., Böckler D., Böhm M., Brückmann H., Debus E.S. (2019). Angioplasty in asymptomatic carotid artery stenosis vs. endarterectomy compared to best medical treatment: One-year interim results of SPACE-2. Int. J. Stroke.

[29] Rosenfield K., Matsumura J.S., Chaturvedi S., Riles T., Ansel G.M., Metzger D.C., Wechsler L., Jaff M.R., Gray W., ACT I Investigators (2016). Randomized Trial of Stent versus Surgery for Asymptomatic Carotid Stenosis. N. Engl. J. Med..

[30] Steinvil A., Sadeh B., Arbel Y., Justo D., Belei A., Borenstein N., Banai S., Halkin A. (2011). Prevalence and predictors of concomitant carotid and coronary artery atherosclerotic disease. J. Am. Coll. Cardiol..

[31] Subherwal S., Bhatt D.L., Li S., Wang T.Y., Thomas L., Alexander K.P., Patel M.R., Ohman E.M., Gibler W.B., Peterson E.D. (2012). Polyvascular disease and long-term cardiovascular outcomes in older patients with non-ST-segment-elevation myocardial infarction. Circ. Cardiovasc. Qual. Outcomes.

[32] Aboyans V., Ricco J.B., Bartelink M.E.L., Björck M., Brodmann M., Cohnert T., Collet J.P., Czerny M., De Carlo M., Debus S. (2018). 2017 ESC guidelines on the diagnosis and treatment of peripheral arterial diseases, in collaboration with the European society for vascular surgery (ESVS). Eur. Heart J..

